# Remedy for Dandruff

**Published:** 1877-01

**Authors:** 


Remedy for Dandruff.—The *American Journal of Pharmacy* says: A French physician recommends to apply a solution of chloral hydrate containing five per cent. of the latter, by rubbing from one-half to one ounce into the scalp by means of a sponge, and repeating it every morning. A slight burning sensation and reddening of the scalp occurs, disappearing after two minutes. If the hair has fallen off in consequence of the dandruff, it will be renewed in about a month.

